# Predicting a small molecule-kinase interaction map: A machine learning approach

**DOI:** 10.1186/1758-2946-3-22

**Published:** 2011-06-27

**Authors:** Fabian Buchwald, Lothar Richter, Stefan Kramer

**Affiliations:** 1 Institut für Informatik, Technische Universität München, Boltzmannstr. 3, 85748 Garching bei München, Germany

## Abstract

**Background:**

We present a machine learning approach to the problem of protein ligand interaction prediction. We focus on a set of binding data obtained from 113 different protein kinases and 20 inhibitors. It was attained through ATP site-dependent binding competition assays and constitutes the first available dataset of this kind. We extract information about the investigated molecules from various data sources to obtain an informative set of features.

**Results:**

A Support Vector Machine (SVM) as well as a decision tree algorithm (C5/See5) is used to learn models based on the available features which in turn can be used for the classification of new kinase-inhibitor pair test instances. We evaluate our approach using different feature sets and parameter settings for the employed classifiers. Moreover, the paper introduces a new way of evaluating predictions in such a setting, where different amounts of information about the binding partners can be assumed to be available for training. Results on an external test set are also provided.

**Conclusions:**

In most of the cases, the presented approach clearly outperforms the baseline methods used for comparison. Experimental results indicate that the applied machine learning methods are able to detect a signal in the data and predict binding affinity to some extent. For SVMs, the binding prediction can be improved significantly by using features that describe the active site of a kinase. For C5, besides diversity in the feature set, alignment scores of conserved regions turned out to be very useful.

## Background

The question whether two molecules (a protein and a small molecule) can interact can be addressed in several ways. On the experimental side, different kinds of assays [1] or crystallography are applied routinely. Target-ligand interaction is an important topic in the field of biochemistry and related disciplines. However, the use of experimental methods to screen databases containing millions of small molecules [2] that could match with a target protein, for instance, is often very time-consuming, expensive and error-prone due to experimental errors. Computational techniques may provide a means for speeding up this process and making it more efficient. In particular in the area of kinases, however, docking methods have been shown to have difficulties so far [3] (Apostolakis J: Personal communication, 2008). In this paper, we address the task of interaction prediction as a data mining problem in which crucial binding properties and features responsible for interactions have to be identified. Note that this paper is written in a machine learning context, hence we use the term "prediction" instead of "retrospective prediction" that would be used in a biomedical context.

**Figure 1:**
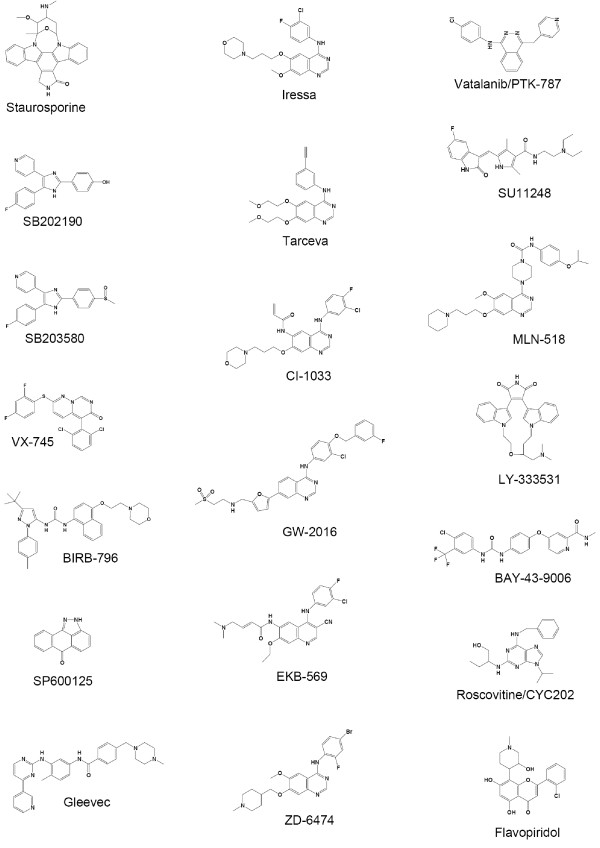
**Training set inhibitors**. Structures of the 20 inhibitors that were subject of our study [7].

In the following, we focus on protein kinases and kinase inhibitors. Protein kinases have key functions in the metabolism, signal transmission, cell growth and differentiation. Since they are directly linked to many diseases like cancer or inflammation, they constitute a first-class subject for the research community. Inhibitors are mostly small molecules that have the potential to block or slow down enzyme reactions and can therefore act as a drug. In this study we have 20 different inhibitors with partially very heterogeneous structures (see Figure 1).

We developed a new computational approach to solve the protein-ligand binding prediction problem using machine learning and data mining methods, which are easier and faster to perform than experimental techniques from biochemistry and have proven successful for similar tasks [4-6]. In summary, the contributions of this paper are as follows: First, it uses both kinase and kinase inhibitor descriptors at the same time to address the interaction between small heterogeneous molecules and kinases from different families from a machine learning point of view. Second, it proposes a new evaluation scheme that takes into account various amounts of information known about the binding partners. Third, it provides insight into features that are particularly important to achieve a certain level of performance.

This paper is organized as follows: In the following sections, we first present the methods and datasets we used, then we give a detailed description of variants of leave-one-out cross-validation to measure the quality of predictions, present the experimental results and finally draw our conclusions.

## Materials and methods

### Data

This section introduces the Ambit Biosciences' dataset [7] that provides us with class information for our classification task. From the dataset we define a two class problem by assigning to each kinase inhibitor pair "binding" or "no binding" according to the measured affinities of interaction read out by quantitative PCR. This dataset is obtained by ATP site-dependent competition binding assays and represents the first approach to mass screening of protein kinases and inhibitors. Table 1 shows overview statistics concerning the size and the class distribution of the dataset. Table S1 in Additional File 1 shows how often an inhibitor binds to a certain group of kinases (group in a phylogenetic meaning). It can be clearly seen that nearly all inhibitors bind to several kinase groups. This means that there is generally no kinase group to which an inhibitor binds consistently. The kinase and inhibitor data, its corresponding features, and the binding matrix is available on the web [8].

**Table 1:** Characteristics of the used dataset

	Ambit Biosciences
number of kinases	113

number of inhibitors	20

number of pairs	2260

number of bindings	597 (26.4%)

number of no-bindings	1663 (73.6%)

For assessing the specificity of protein kinases for inhibitors, Ambit applied ATP site-dependent competition binding assays that directly and quantitatively measure the binding of inhibitors to the ATP binding site of kinases.

### General approach

According to the SAR (Structure Activity Relationship) paradigm that activity is related to structure, we put the focus on features that describe the structure of molecules, that are leading to certain structures (sequence-based features) or that determine the chemical environment of the active site of a kinase or the molecule as a whole with respect to the inhibitors. Features from further categories may also give hints whether an interaction can take place, like information about the similarity of molecules, e.g., alignment scores or other phylogenetic features.

### Feature generation

In the following, we will describe the features chosen to describe the kinases and inhibitors.

#### Feature generation for kinases

One class of features for the kinases represent sequence-based features. These features are derived from consecutive patterns of single amino acids. However, only frequent patterns are regarded as interesting, since only those can be informative for prediction. Active sites, for instance, are usually highly conserved and of special interest since inhibitors bind to that region. We scanned the PROSITE database (release 51.2) for PROSITE patterns that match our protein kinase sequences. These patterns are characteristic clusters of residue types occurring over a rather short section of a protein sequence. For the generation of further frequent patterns we implemented Agrawal and Srikant's APriori algorithm [9] with minor modifications. During the levelwise search [10] that enables us to find all frequent patterns, we count per example: multiple occurrences within a kinase are only counted once. As a refinement operator to generate patterns for the next level, we used pattern merging. In a pattern we allow wildcards, however, their number is retricted to two in order to reduce the search space. We excluded wildcards at the beginning and at the end of patterns, since they do not carry any significant biological information. The sequence-based features are represented in bitvectors that indicate whether a sequence is present or absent in a kinase.

Each position in a bitvector corresponds to a sequence where "1" indicates presence and "0" absence. Another class of features are phylogeny-based features. We extract available phylogenetic information about the kinases from KinBase [11] since a closer phylogenetic relationship implies a higher sequence similarity and thus also a higher similarity in the overall 3D structure - especially at conserved sites like the active center. We grouped the kinases into Serine/Threonine and Tyrosine kinases and made a finer division into kinase groups and kinase families. The phylogeny-based information is presented in nominal form, e.g. the phylogenetic feature "group" has several categories like AGC, CAMK or CK1.

Other phylogenetic features are directly derived from kinase sequence alignments. We implemented three different types of alignment procedures. First, a global alignment algorithm [12] was used that aligns two amino acid sequences over their full length. The protein kinases we investigated differ enormously in length, reaching from 275 to 1,607 amino acids, and therefore many gaps requiring to be introduced. This may obscure existing evolutionary relationships making therefore the alignment scores less useful. To overcome this problem, first we applied CLUSTALW [13] to get a multiple sequence alignment (MSA), from which we selected highly conserved sequence stretches (frames) where each frame must satisfy the following three criteria:

- At least one position in a frame must be highly conserved. A highly conserved position is a residue in which one amino acid occurs in more than 100 out of the 113 cases.

- The frame border is at most five amino acids away from a highly conserved position.

- A highly conserved position must be part of the active center.

For each kinase pair, each frame is matched with the corresponding frame from the second sequence and scored according to the scoring matrices (see below). The scores for the frame pairs are summed up to get an overall score for all conserved regions, whereas a higher score should indicate a more similar active center and more similar binding properties. To calculate the scores, we implemented two different techniques. First, we just cut out the amino acid sequences of the frames from the MSA and scored it without any further modification. Second, we realigned the cut out frames before calculating the total score. For all alignment procedures we used PAM120 and Blosum62 as substitution matrices with uniform costs for gap opening and gap extension. The alignment-based features are represented with a 113-dimension vector where each dimension or postition represents a numeric alignment score.

Additionally, we also use single residues which contribute to the active site and inhibitor binding as position specific features. These features' values are either the respective amino acid or the physico-chemical class of the amino acid. In Table 2, the features, the number of features, and the feature type in each group used in our study for describing the kinases are summarized.

**Table 2:** Summary of different features of kinases used in our study

Short-hand	Full Name	# Features	Feature Type
*STTK*	Serine/Threonine, Tyrosine Kinases	1	nominal

*Summary* Partitioning into Serine, Threonine and Tyrosine kinases			

*PC*	Phylogenetic Clustering	2	nominal

*Summary* Partitioning into kinase groups and kinase families			

*PRO*	PROSITE patterns	12	numeric

*Summary* Find PROSITE patterns in the kinases			

*Apri*	Apriori patterns	14	numeric

*Summary* Find frequently occurring amino acid sequence patterns			

*glAli*	global alignment scores	113	numeric

*Summary* Calculate global alignment scores for all pairs of kinases			

*locAli*	local alignment scores	113	numeric

*Summary* Calculate local alignment scores for all pairs of kinases			

*PSF*	Position Specific Features	98	nominal

*Summary* Use amino acids at the active center directly as features			

*abPSF*	abstract Postition Specific Features	98	nominal

*Summary* Use amino acid classes at the active center directly as features			

#### Feature generation for inhibitors

For the description of the inhibitors we used features based on their 2D structures, preferred binding partners (primary targets) and binding patterns. We visually clustered the inhibitors by simply looking at their 2D structure so that inhibitors with similar shapes are grouped together (see Table 3). Primary targets are kinases for which an inhibitor shows a highly preferred binding compared to other kinases. In this context, "primary target" concerns kinases in general and is not restricted to the kinases under consideration in this paper. Binding patterns represent the binding behavior of an inhibitor to a set of kinases and may serve also as features since similar properties on known targets give hints to binding properties on unknown targets. Therefore, we implemented a *k*-nearest-neighbor method (KNN) to detect each inhibitor's *k *nearest neighbors. In this study we used *k *= 3. The calculation is based on data from Ambit's binding matrix. Note that this calculation is only possible in the "soft case" evaluation (to be presented below) since only in that case all the information of a test kinase (respectively inhibitor) relative to all training inhibitors (respectively kinases) is given. As a distance measure, we used a function counting the number of common bindings of two inhibitors *x_i_*, *x_j _*(*c*), and the more complex Tanimoto coefficient (1) that counts besides (*c*) the number of bindings of inhibitor *x_i _*to a kinase (*a*) and the number of bindings of inhibitor *x_j _*to the same kinase (*b*):(1)

**Table 3:** Summary of different features for the inhibitors used in our study

Short-hand	Full Name	# Features	Feature Type
*PT*	Primary Target	1	nominal

*Summary* Determine for which kinase(s) an inhibitor shows a preferred binding			

*MS*	2D Molecular Structure	1	nominal

*Summary* Clustering of inhibitors due to their 2D structure *Cluster*_1 _= {*SB*202190, *SB*203580} *Cluster*_2 _= {*CI*-1033, *EKB*-569, *MLN*-518} *Cluster*_3 _= {*Staurosporine; LY*-333531} *Cluster*_4 _= {*Roscovitine; Flavopiridol; BIRB*-796} *Cluster*_5 _= {*SU *11248*; BAY*-43-9006*; ZD*-6474*; Gleevec*, *GW*-2016, *Iressa*, *Tarceva*, *V X*-745, *V atalanib*, *SP*600125}			

*FTs*	Free Trees	78	numeric

*Summary* Determine frequently occurring acyclic substructures as structural features			

*KNN*	KNN clustering	20	numeric

*Summary* Detect each inhibitor's *k *nearest neighbors			

*CF*	Chemical Features	15	numeric

*Summary* Calculate various chemical features with JOELib2			

*GF*	Geometric Features	5	numeric

*Summary* Calculate various geometric features			

*P*	Pharmacophores	50	numeric

*Summary* Calculate 3-point pharmacophores			

To describe the inhibitor structures, we applied the graph mining tool Free Tree Miner (FTM) [14]. With this tool, the 2D structure representations of the inhibitors are mined for frequently occurring acyclic substructures. Such substructures can describe, for instance, a hydrophobic group in an inhibitor important for bindings or an extended region that would exclude small active sites of kinases as binding partners due to steric hindrance. To avoid the exclusion of probably important substructures right from the beginning, we set the minimum frequency threshold rather low to 10%.

Additionally, we also calculated geometric features of the inhibitors from 2D data like their diameter, length and width that might prevent kinase binding due to steric hindrance.

Besides this, various chemical features determine whether or not a binding at the active site can take place. For the calculation of such features, we applied the cheminformatics library JOELib2 [15]. In this way, we obtained the following physicochemical features: XlogP, molecular weight, hydrogen bond acceptor/donor count, rotatable bond count, tautomer count and topological polar surface area. All these features are suitable for building basic structure-activity relationship (SAR) models [16]. We also described the inhibitors with pharmacophores. A pharmacophore is, in general, a 3D substructure of a molecule that is meaningful for its medical activity. It can be seen as an abstraction of the molecular structure to a usually small number of key features that contribute to the majority of the activity together with their geometric arrangement that is represented by pairwise distances. For the actual calculation of the pharmacophores we, only for simplicity, used the 2D information of the inhibitors. We calculated so-called 3-point pharmacophores [17] for our set of inhibitors. Such pharmacophores consist of three essential atoms (negatively or positively charged atoms, acceptor or donor atoms) and their distances in space. We calculated all 3-point pharmacophores, sorted the atoms lexicographically in order to avoid duplicates, and used the atoms as well as their (discretized) distances as features. Table 3 summarizes the features, the number of features and the feature type in each group that we used for describing the inhibitors.

The instances consisting of kinase-inhibitor pairs are represented by concatenating kinase and inhibitor feature vectors, i.e. each kinase is concatenated with each inhibitor. Formally, this can be stated as(2)

where *K_i _*represents the feature vector of the *i*th kinase and *I_j _*the feature vector of the *j*th inhibitor.

### Feature selection/reduction

Feature selection techniques attempt to determine appropriate features that can discriminate well between classes. Feature sets that are too larger may contain many uninformative features leading to overfitting or a decrease in prediction accuracy or efficiency. On the other hand, feature sets which are too small may not contain enough information to determine the target class and may cause underfitting.

The feature sets generated by APriori usually contain many solution patterns which are redundant or less useful as they are too small (i.e., strings/trees of length one). Such elements can be removed, and the size of the complete solution set can be reduced significantly, e.g. by computing so-called *border elements *[18], i.e., the most specific patterns that are still solutions. We calibrated Free Tree Miner to solely output border elements. Apriori was implemented to output only features that are border elements and larger than a user defined size threshold. Finally, we used in our study 14 sequence-based apriori features and 78 free trees (see Tables 2, 3).

### Classification

For classification, we used standard schemes like decision tree (C5) and large margin (SVM) learning methods. C5 [19] is commercial improvement of C4.5 [20] written in C and popular for its efficiency. For the SVM [21], we used Weka's [22] implementation of Sequential Minimal Optimization (SMO) [23]. We tested three kernels (linear (E1), quadratic (E2) and radial basis function (RBF)) with Weka's default parameter setting including the cost factor *C *1.0. A higher *C *slows down the running time of the classifiers. A *C *of 0.1, however, renders the RBF kernel SVM to a majority class predictor. For an SVM with a linear kernel the opposite is true, but it performs in all cases on a lower level. The performance of the quadratic kernel SVM remains nearly the same on the test data, on the training data, however, a smaller *C *decreases the preditive power. For C5, the main task consists of finding the best pruning options to control overfitting. C5 provides the option to prune with confidence intervals and with a minimum support of training instances that must be covered by each leaf of the tree. We used C5's default settings, with a pruning confidence factor of 25% and a minimum support in each leaf of 2. Subsequently, global pruning can be used to optimize the tree's performance further.

Note that for C5, continuous or numeric features are discretized using standard procedures [20]. For SVMs, nominal features are transformed to "binary numeric" using Weka's standard filter *NominalToBinary *[22,24]. All features used within SVMs are normalized by the Weka workbench by default. The kernels we applied are constructed out of all these normalized features.

## Results

### Evaluation

We use leave-one-out cross-validation (LOOCV) to evaluate our classification results. LOOCV may appear uncommon, at first sight, in this setting with 2260 instances since it is generally recommended (along with the bootstrap) for smaller datasets. This is because a smaller number of folds would result in an even larger variance. LOOCV is known to deliver estimates with a small bias, whereas the variance can be high. However, with more than 2000 instances, the training sets do not vary a lot; therefore, even the variance is low in this case. Usually, ten times ten-fold cross-validation is preferred on such datasets for practical reasons, to avoid the excessive running times of LOOCV. However, we wanted to test the "purest" setting and also obtain maximally unbiased error estimates. Finally, it should be clear that the proposed evaluation variants can easily be extended towards regular k-fold cross-validation, by leaving out pairs of sets of kinases and sets of inhibitors in turn. To evaluate the quality of a model, we used three established performance measures: In-/correctly classified instances, recall and precision:(3)(4)(5)(6)(7)

Note that (4) is also known as Sensitivity and True Positive Rate (TPR), (5) as Selectivity and Positive Predicted Value (PPV), (6) as Specificity and True Negative Rate (TNR), and (7) as Negative Predicted Value (NPV).

In the following, we will present a new way of evaluating classifiers in the present setting, and give an overview of four different variants of LOOCV applied here. Since we aim for predictions for pairs of kinases and inhibitors, different amounts of information may be available for the two potential binding partners.

#### The hard and the soft case

**Figure 2:**
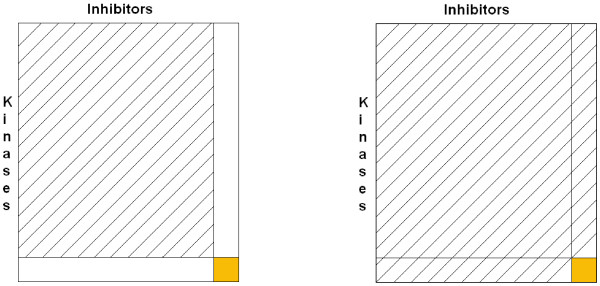
**Hard and soft case of LOOCV**. Illustration of the hard (left) and the soft (right) case of LOOCV.

Figure 2 shows the two extreme variants of our different implementations of LOOCV. The left-hand side of the figure shows the "hard case", in which no information about the test kinase and the test inhibitor is allowed in the training dataset. This would, for instance, represent the scenario in which a binding prediction is performed for a completely unknown pair of a kinase and an inhibitor. In the "soft case" (see the right-hand side of Figure 2), however, all information about the test kinase and the test inhibitor is already known in the training set - except for the pair itself to be predicted.

#### The mixed and the mixed-mixed case

**Figure 3:**
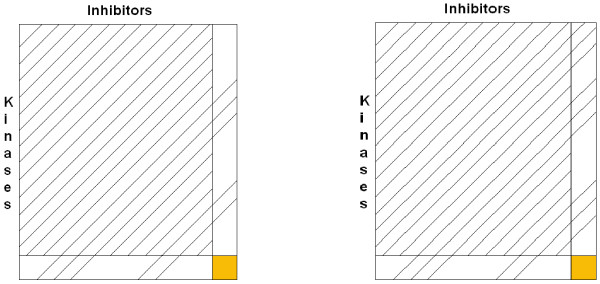
**Mixed and mixed-mixed case of LOOCV**. Illustration of the mixed (left) and the mixed-mixed (right) case of LOOCV.

The two cases between the extreme variants of our different implementations of LOOCV are shown in Figure 3. The left side of the figure illustrates the "mixed case", in which the equal percentage of information on the test kinase and the test inhibitor are put into the training set. This means that a certain random fraction from the test kinase and the same random fraction from the test inhibitor is put into the training set. To give an example, if we use 50% of the test inhibitor information, we put 10 kinase-inhibitor pairs in the training set where the inhibitor in the pair must be the inhibitor to be predicted. For the kinase the same holds, but 50% make up 57 pairs. On the right side, the "mixed-mixed case" is illustrated, in which the training dataset contains information on the test kinase and test inhibitor in an unequal proportion. This represents the situation in which experimental information concerning the binding patterns of a certain test kinase to the inhibitors is partly available. For the test inhibitor, the same holds, but in a different proportion.

#### Results for different feature sets

We start with the results from the soft case evaluation. In the following, we show how different feature sets (see Table 4) affect the performance of the classifiers. The overall plan of the experiments was (1.) to start with a set of base features for both kinases and inhibitors, (2.) to refine the representation of kinases in the next step, and after that (3.) to refine the representation of inhibitors. For the representation of kinases, we add alignment-based features, then position-specific features, and ultimately both alignment-and position-specific features. For the representation of inhibitors, we start with the base features and then add further descriptors (CF, GF and P in the table) in a final refinement step.

**Table 4:** This table indicates which features are contained in which feature sets

	FS1	FS2	FS3	FS4(C5)	FS4(SVM)	FS5	FS6(C5)	FS6(SVM)	FS7(C5)	FS7(SVM)
PT	X	X	X	X	X	X	X	X	X	X
MS	X	X	X	X	X	X	X	X	X	X
FTs	X	X	X	X	X	X	X	X	X	X
KNN	X	X	X	X	X	X	X	X	X	X

CF									X	X
GF									X	X
P									X	X

STTK	X	X	X	X	X	X	X	X	X	X
PC	X	X	X	X	X	X	X	X	X	X
PRO	X	X	X	X	X	X	X	X	X	X
Apri	X	X	X	X	X	X	X	X	X	X

glAli		X		X			X		X	
locAli			X	X			X	X	X	X
PSF					X	X	X	X	X	X
abPSF						X	X	X	X	X

This table indicates which features are contained in which feature sets. PT: Primary Targets, MS: 2D Molecular Structure, FTs: Free Trees, KNN: KNN clustering, CF: Chemical Features, GF: Geometric Features, P: Pharmacophores, STTK: Partitioning in Serine-, Threonine and Tyrosine Kinases, PC: Phylogenetic Clustering, PRO: PROSITE patterns, Apri: APriori patterns, glAli: global alignment scores, locAli: local alignment scores, PSF: Position Specific Features, abPSF: abstract Position Specific Features. The upper part of the table describes the chemical features, the lower part the biological features. In the left part of the table (from FS1 to FS6), the description of the kinases is optimized (testing combinations of alignment-based and position-specific features). In the right part (FS7), the chemical representation is further optimized by additional descriptors.

In preliminary experiments, we evaluated the individual performance of feature groups (Table 5). Here the features for kinases perform very similarly, all in a range between 73% and 74% (for C5), whereas the features for inhibitors differ in their performance: the predictive accuracy of *CF*, *GF*, *KNN*, *FTs*, and *P *range between 79% and 80% (again for C5), with the remaining two feature groups (*PT *and *MS*) lagging behind. Results for SVMs are mostly comparable (see Table 5).

**Table 5:** Comparison of prediction accuracies for single feature groups

Comparison of prediction accuracies for single feature groups on the test set															
**FS**	**PT**	**MS**	**CF**	**GF**	**KNN**	**FTs**	**P**	**STTK**	**PC**	**PRO**	**Apri**	**glAli**	**locAli**	**PSF**	**absPSF**

C5	73.6	75.6	79.3	79.3	79.3	79.3	79.3	73.6	73.6	73.6	73.6	74.0	74.0	73.3	73.3

SVM	73.6	75.6	79.3	73.6	76.8	79.3	79.3	73.6	73.5	73.1	73.1	72.7	73.6	72.3	72.3

Comparison of prediction accuracies of C5 without global pruning and SVMs with the quadratic kernel for single feature groups on the test set. For a description of the abbreviations of the feature sets see Table 4.

**Figure 4:**
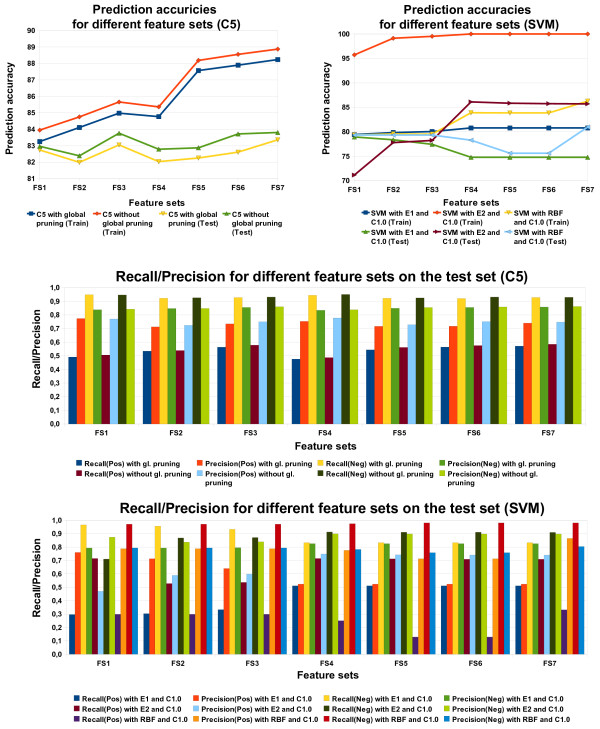
**Performance on different feature sets (soft case)**. Prediction accuracies, recall and precision for different feature sets from C5 and Support Vector Machines with different parameter settings (soft case).

Figure 4 shows the prediction accuracies for different feature sets for both SVMs and C5 that were run with different parameter settings. In all cases C5, without the option "global pruning", outperforms the other variant with global pruning. Compared to the SVM, it is extremly fast and handles large feature sets well concerning runtime and memory.

For C5, the usage of global alignment scores as features (FS2) reduced the prediction accuracy on test data significantly. This may be explained by the fact that these scores take into account the complete amino acid sequence, whereas only a small part constitutes the active center and is therefore important for the binding to an inhibitor. These non-informative sequences clearly outweigh the informative ones, and so they obscure the information and make the scores for global alignments less useful. Alignment scores of extracted conserved regions perform clearly better on the training and test set. In this case it is particularly remarkable that cut out and realigned conserved regions (FS3) perform 1.6% better than without realigning on the test set.

The most difficult prediction task for the applied classifiers was the correct prediction of a binding between an inhibitor and a kinase. For all different feature sets, C5 as well as SVMs have the lowest values for the recall of the positive class (Figure 4). Particularly conspicuous are the extremely low values for the recall of the positive class of SVMs with an RBF and a linear kernel. For C5, feature sets 3, 6 and 7 clearly show the best positive recall (and prediction accuracy) for the test data. Particular attention should be paid to FS3, which comprises, besides the basic feature set, only local alignment scores. This indicates that local alignment scores are very suitable for making predictions with C5. The negative recall is relatively constant for all feature sets. However, the tradeoff between positive and negative recall is visible.

Comparing FS1 with FS3, or FS4 with FS7, shows that a higher positive recall leads to a lower negative recall. A combination of global and local alignment scores, as well as a combination of position specific features with abstract position specific features, degrades the predictivity. Only when we combined alignment score features (FS4) with position specific features (FS5) to feature set 6, the prediction accuracy increases significantly on the training and test set. This combination is then further improved by adding chemical features leading to the best prediction accuracy we obtained from C5 on the test set. This success can largely be attributed to the use of chemical features and the diversity of the features.

With SVMs, we used global alignment scores as features (FS2) only once, since they slow down the computation enormously and when combined with other feature sets, do not contribute to an increase in the prediction accuracy on the test set. However, we tested the influence of using position specific features with (FS5) and without abstractions (FS4), where the use of abstract position specific features did not show an improvement of the prediction accuracy.

##### Comparison of different kernels in SVMs

The differences between the kernels are clearly observable from Figure 4. The quadratic kernel performs with higher success than the linear and RBF kernel for all feature sets except for FS1 and FS2 for both kernels and FS3 for the RBF kernel on the test set. On the training set, this fact must be mainly attributed to overfitting. On the test set, the best results are obtained with feature set 4. This indicates that SVMs with a quadratic kernel work best with position specific features. This may be due to the fact that we described here for the first time the active site of a kinase with position specific features. An addition of further features does not lead to an increase in the predictive power for both training and testing.

For the linear and RBF kernel things are different. A larger amount of features increases the predictivity on the training data set but harms it on the test data set except for FS7 with the RBF kernel. From Figure 4 it is evident that the recall for the negative class normally drops from feature set 1 to feature set 7 for the linear kernel. The reason for this may be that SVMs are not able to predict the "binding" class with features that do not discriminate immediately between the classes. Hence, SVMs mostly predict the majority class "no-binding", leading to a high negative recall. But with increasing ability to discriminate between the classes, more bindings are predicted correctly, leading to an increase in the recall for the positive class. On the test set this is accompanied by a decrease in the recall for the negative class and an increase in its corresponding precision (Figure 4). For the RBF kernel we obtain the best predicitivity as well as the highest positive and negative recall with FS7. The chemical features seem to be the most decisive ones with respect to the RBF SVM. From feature set 1 to feature set 6 the negative recall and precision remain relatively constant. The positive recall and precision, however, decrease significantly.

##### Performance on random feature sets

**Figure 5:**
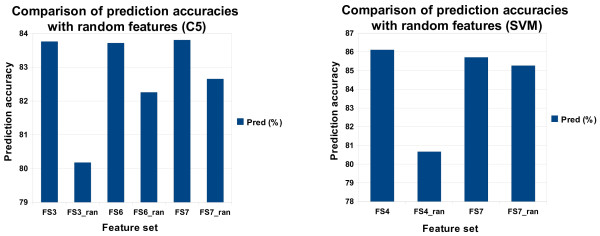
**Comparison of prediction accuracies with random features**. Comparison of prediction accuracy for different feature sets including random features.

We also tested the performance on features sets with random feature values. For feature set 3, we assigned random integers (FS3_ran) to all local alignment score values where a random integer must be in the range between the smallest and largest value of the true values. Results for C5 and SVMs with a quadratic kernel are shown in Figure 5. As expected, the usage of random features harms performance. For C5, it is visible that for feature set 3 the drop of the performance is larger than for feature sets 6 or 7. The same holds for SVMs with respect to feature set 4 and 7. This can be explained by the fact that the amount of random features for feature set 3 is much higher than for feature set 7, for instance, where random values are assigned to three rather small feature groups (chemical, geometrical and pharmacophoric features). Particularly, the worse performance of the random feature sets 6 and 7 indicates that the sheer size of a feature set does not neccessarily contribute to a better performance through chance correlation or overfitting, but that the diversity of a feature set is the factor positively impacting predictivity.

#### Results for the hard and the soft case evaluation strategy

**Figure 6:**
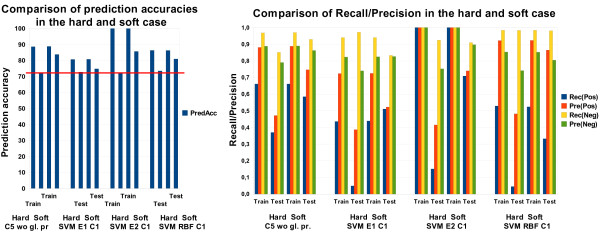
**Performance comparison of the hard and the soft case**. Comparison of the prediction accuracy and recall/precision in the hard and the soft case.

All the results in this section are based on feature set 7. C5 shows only slight, nonsignificant differences between the hard and the soft case concerning the training data, however, on the test data there are large differences (Figure 6). In the hard case, recall and precision values for the positive class are very low, which indicates that the classifier is not good at identifying kinase and inhibitor features responsible for binding. From the hard to the soft case, these two values clearly increase. In contrast, there is only a small drop in the recall and the precision for the negative class. As for C5, the prediction accuracy on the test and the training set of SVMs (with a linear, quadratic or RBF kernel and cost factor C = 1.0) always increases from the hard to the soft case. In the hard case, there are even worse results regarding the recall for the positive class (5.0% recall). The quadratic kernel clearly overfits on the training data.

In some hard cases for different feature sets (not shown here), especially for small feature sets with predominantly features that are not able to discriminate between classes, C5 as well as SVMs are performing as good as a majority class classifier since they predict everything as non-binding. For more complex feature sets like in the cases shown here, C5 and the SVM classifiers with a linear or quadratic kernel are slightly worse than a majority class classifier (red line in Figure 6) that would reach 73.6%. SVMs with an RBF kernel, however, perform slightly better although the recall for the positive class is very low. However, in this case, this is compensated by a high precision for the positive class as well as a high recall for the negative class (see Figure 6).

In the soft case, we compare our prediction accuracy results with a simple baseline classifier that calculates the probabilities for a binding from the binding matrix, not taking into account any information about the molecules. This is only possible in non-hard cases, since the information how often a test kinase/inhibitor binds to a training set inhibitor/kinase is directly taken into account. More precisely, the simple baseline classifier calculates, separately, the probabilities *p_kin_*(*b*) and *p_inh_*(*b*) of a test kinase/inhibitor binding on the training set. Subsequently, these probabilities are multiplied and it is determined whether the product is greater than the threshold *θ *that was optimized empirically:(8)

A smaller value than *θ *results in a "no-binding" prediction, otherwise "binding" is predicted. This simple classifier is able to reach 78.5% prediction accuracy without any knowledge about the kinases or inhibitors except their binding patterns. It is clearly better than a majority class classifier, but worse than models that consider additional information about the molecules.

**Figure 7:**
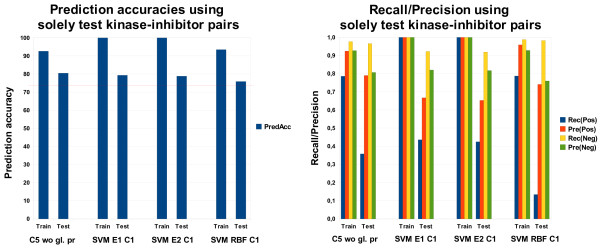
**Performance using solely test kinase-inhibitor pairs**. Comparison of prediction accuracy and recall/precision using solely test kinase-inhibitor pairs in the training set.

The difference between the hard and the soft case are the kinase-inhibitor pairs in the training set that contain either the test kinase or the test inhibitor. The performance improvement of the classifiers must be attributed to these pairs. Figure 7 shows the results if we remove all kinase-inhibitor pairs from the training set which do not contain the test kinase or the test inhibitor. On the training set (consisting of 131 instances or kinase-inhibitor pairs), a strong performance can be observed. Compared to the soft case (see Figure 6 soft case), however, the results on the test set, disregarding SVMs with a linear kernel, show a clear performance loss if we remove kinase-inhibitor pairs from the training set which contain neither the kinase nor the inhibitor to be predicted. The usage of test kinase and test inhibitor information, solely, is not sufficient to obtain reasonable results. This means that the applied machine learning methods require the other pairs in order to generalize well.

#### Results for the mixed and the mixed-mixed case

**Figure 8:**
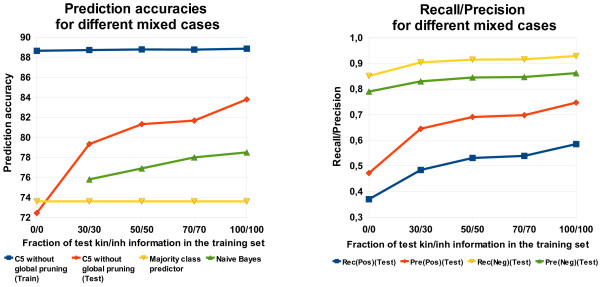
**Performance comparison of different mixed cases (C5)**. Comparison of prediction accuracy and recall/precision for different mixed cases (C5 without global pruning).

All the results in this section are based on predictions of C5 that was run on feature set 7 without global pruning. Figure 8 shows the prediction accuracies of three mixed cases, the soft and the hard case, a simple baseline classifier and a majority classifier, as well as the recall and precision values for our predictions. The results for the mixed cases and the simple baseline classifier are obtained by averaging the results from ten runs of C5 with identical parameter settings. Note that we took randomly a certain fraction of test kinase/inhibitor information in the training set. This means that the results in each run can be slightly different. Hence, it is necessary to conduct several, in our case 10, experiments with the same parameter setting and average the results in order to take these variations into account.

The performance on the test set is strongly influenced by the usage of different fractions of the test kinases and the test inhibitors in the training set. The performance on the training data, however, is nearly independent of it (see Figure 8). The strong increase in performance from the hard case to the first mixed case (30/30) indicates the importance of the information about the test kinase and the test inhibitor. The usage of information from the test kinase and the test inhibitor leads to a substantial increase in the recall and the precision for the positive and the negative class. The classifier learns to discriminate better between the classes and thus to predict a correct binding more often without losing performance on the negative class.

**Figure 9:**
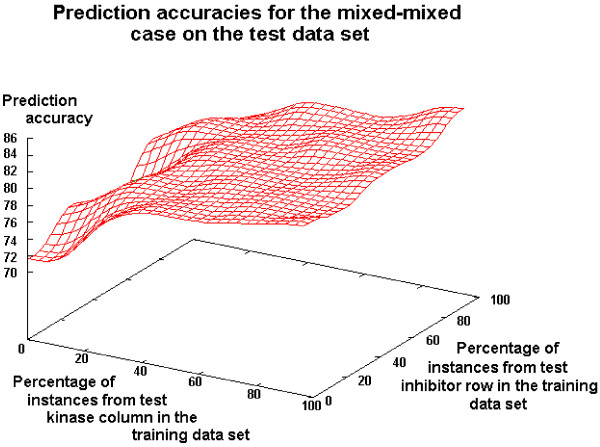
**Performance comparison of different mixed-mixed cases (C5)**. Comparison of prediction accuracy for the soft, hard, mixed and mixed-mixed cases (C5 without global pruning).

Further results (not shown here) reveal only slight differences in the training set performance for the mixed-mixed case. Here, the classifier performs clearly independent of the number of instances from the test kinase and the test inhibitor in the training set. On the test set, however, there is great variability in the prediction accuracy of different mixed-mixed cases. First, we analyze the performance if a percentual amount of information on the test kinase and the test inhibitor is added to the training set. Note that this means that actually more information on the test kinase is added since the dataset consists of more kinases than inhibitors. Second, we analyze the case in which equal information on the test kinase and the test inhibitor is added to the training set, i.e. if 10 pairs consisting of the test kinase and 10 different training set inhibitors are added, then 10 pairs of the test inhibitor and 10 different training set kinases also have to be added. Further note that the training set without test molecule information can be seen as a reference. This reference represents the hard case. For both variants, it is tested how information on test kinases and test inhibitors in the training set can improve the performance compared to the reference. From Figure 9, the worst and the best values are obtained in the hard and the soft case, respectively. The same holds for the corresponding cases in Table 6 (0/0 and 19/19). Results with percentual and absolute test molecule information are similar. For the dataset under investigation, it can clearly be seen that the information about the kinase test molecule is more important than that of the tested inhibitor. If no information from the test inhibitor and only little information from the test kinase is in the training set, the prediction accuracy increases significantly. In contrast, no information from the test kinase and little information from the test inhibitor leads to a remarkably lower increase in the prediction accuracy (see Figure 9 and Table 6). This can be clearly seen, for instance, in Table 6 for the cases 10/0 and 0/10.

**Table 6:** Comparison of prediction accuracies for some mixed-mixed cases (on an absolute basis) (FS7)

Comparison of prediction accuracies of C5 for some mixed-mixed cases on the test set (on an absolute basis)									
**kin/inh**	**0/0**	**10/0**	**19/0**	**0/10**	**0/19**	**10/10**	**10/19**	**19/10**	**19/19**

C5	72.4	77.5	78.6	73.5	73.9	78.9	79.1	79.4	79.5

Comparison of prediction accuracies for C5 for some mixed-mixed cases on the test set (on an absolute basis). Results are obtained with feature set 7.

We benchmarked our kinase inhibitor binding prediction approach for the mixed-mixed cases and the soft case with a majority class classifier as well as with a simple baseline classifier (Table 7). The more informed classifier, C5, performs in all cases, except one, better than both reference classifiers. Mostly, a clear performance improvement with respect to prediction accuracy can be observed. This means that the feature extraction from the kinases and the inhibitors is beneficial. The same holds for the mixed cases and the soft case.

**Table 7:** Comparison of prediction accuracies for some mixed-mixed cases (on a percentage basis) (FS7)

Comparison of prediction accuracies of different classifiers for some mixed-mixed cases on the test set											
**kin/inh**	**80/20**	**20/80**	**60/40**	**40/60**	**50/0**	**0/50**	**100/0**	**0/100**	**100/50**	**50/100**	**100/100**

C5	81.4	79.4	80.6	80.5	79.6	71.8	82.7	73.9	83.1	81.1	83.8

Bayes	76.1	77.2	76.3	76.7	73.6	73.6	73.6	73.6	77.6	78.2	78.5

Maj.	73.6	73.6	73.6	73.6	73.6	73.6	73.6	73.6	73.6	73.6	73.6

Comparison of prediction accuracies of different classifiers for some mixed-mixed cases on the test set.

Results are obtained with feature set 7.

In summary, the best model achieved 83.8% predictive accuracy with a recall of 0.59 and a precision of 0.75 for the positive class. The most frequently used features in the learned decision tree are position-specific features, local alignment features and the JOELib2 chemical features.

#### Results for an external test set

**Figure 10:**
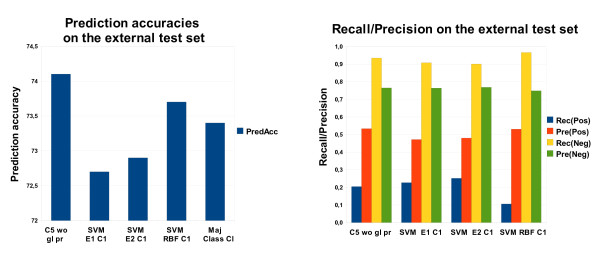
**Performance on the external test set**. Prediction accuracy and recall/precision on the external test set with feature set 7, for both C5 and SVMs.

In addition, the classifiers were tested on an external dataset consisting of 19 kinase inhibitors and 177 protein kinases [25]. It is the result of a later study from Ambit Biosciences and produced in the same way as the dataset described in the section "Data". Note that the original dataset consists of 38 inhibitors and 317 kinases. We removed inhibitors and kinases that are contained in the training set [7] and those where information is missing needed for descriptor calculation. The class distribution of this compiled dataset is similar with 26.6% bindings and 73.4% non-bindings. Testing on an external dataset corresponds to the hard case since neither information about the inhibitors nor information about the kinases is available. The best results on feature set 7 we obtained with C5 without global pruning (prediction accuracy on test set: 74.1%). SVMs with an RBF kernel are also able to outperform a majority class predictor. SVMs with a linear or quadratic kernel, however, perform slightly worse than a majority class predictor (Figure 10). These results represent a clear improvement in comparison to the hard case in the LOOCV setting. Primarily, this improved performance can be explained by structurally similar inhibitors present in the training set, which are not available in LOOCV (see Figure 1).

## Related Work

Kinase inhibitor predictions have been investigated over the past few years. Basically, there exist two approaches. The simpler, and more established one, is to calculate a vectorial representation of both kinase inhibitor and non-kinase inhibitor molecules and using the result with standard machine learning algorithms to predict the probability of a molecule to be a kinase inhibitor. This approach was, for instance, taken by Briem and Günther [26]. In their study, they used a Schering in-house dataset of small molecules encoded by 120 fragment-based Ghose-Crippen descriptors and applied several machine learning techniques (SVMs, artifical neural networks, kNN with GA-optimized feature selection and recursive partitioning) to distinguish between kinase inhibitors and molecules with no reported activity on any protein kinase. Since the original dataset was strongly imbalanced, a ensemble-based sampling procedure was applied to ensure balanced training sets. In the end, 13 training sets were generated for model learning and applied to an independent test set. Briem and Günther analyzed to what extent machine-larning algorithms are capable of learning kinase inhibitor likeness and to compare the different classifiers. Results are reported for each of the 13 individual sample classifiers and for a consensus majority vote of all members of the ensemble. The results show that the latter generally outperforms averaging over the individual models. All four methods exhibited a reasonable discriminative power. Comparing the individual classifiers with respect to standard quality measures, SVMs seem to be the best choice. This is also true for a further compiled test set with significantly different structures.

Xia and colleagues [27] used a modified Naive Bayes classifier to model multifamily and single-target kinase inhibitors. In their study, they used Amgen's CORP datasets (around 200.000 molecules) composed of kinase inhibitors, potential kinase inhibitors, and random drug molecules. To describe the molecules, standard physicochemical features as well as a 2D structural fingerprint were used. To assess the performance of the Bayesian model, the positions of active compounds in ordered scoring lists of the test set were used. The approach was validated by first using an equal proportion of training and test instances (1:1) and second using a much smaller training set (1:9). The results suggest that only 10% of the data are enough to yield a performance nearly as good as if 50% were used. 85% of the active compounds occurred in the top 10% of the ordered molecules. This underlines the power of the Bayesian model which is also confirmed on 172 novel kinase inhibitor compounds from different structural classes that were classified with the 1:9 Bayesian split model. 70% of these new compounds were found in the top 10% and 85% in the top 20% rank-ordered compounds.

Compared to our study, Briem and Günther as well as Xia and colleagues used only information of small molecules (kinase inhibitors, potential kinase inhibitors and random drug molecuels) for predicting the probability of a molecule being a kinase inhibitor. Information about kinases is not considered.

The second, and more difficult approach, is to use features from kinase inhibitors and protein kinases in combination. Weill and Rognan [28] presented a novel low-dimensional fingerprint approach encoding ligands and target properties to mine the protein-ligand chemogenomic space. Kinase inhibitors are represented by standard descriptors, while protein transmembrane cavities are encoded by a fixed length bit string describing pharmacophoric properties of a defined number of binding site residues. Due to the complexity of the cavity, this study is restricted to G protein-coupled receptors (GPCRs) with a homogeneous cavity description. Several machine learning classifiers on two training sets of roughly 200.000 receptor-ligand fingerprints with different definitions of inactive decoys are applied for model learning. Two external test sets of 60 GPCRs were used to validate the models. Experimental results suggest that SVMs with an RBF kernel perform best with respect to a balanced accuracy measure combining true positive and true negative rate. The authors demonstrate that protein-ligand fingerprints outperform the corresponding ligand fingerprints in predicting either putative ligands for a known target or putative targets for a known ligand. They conclude that, with respect to GPCRs, predicting ligands is significantly easier than predicting targets.

Our approach resembles the one of Weill and Rognan in that both kinase and inhibitor information is used for modeling. However, a key difference in Weill and Rognan's approach is the restriction to GPCRs, whereas in this paper, we take a broad spectrum of different kinase families into account and thus are able to make predictions for a larger range of kinases and inhibitors.

## Conclusion

We tackled the prediction task whether a binding between a protein kinase and an inhibitor can take place, given a set of features describing both molecules. We applied and tested a range of data mining and classification tools. Finally, we used both C5 and Support Vector Machines together with three variants of leave-one-out cross-validation to learn and validate concepts of protein kinase inhibitor bindings and the influence of information available about the potential binding partners. The approach performs well in the soft case validation and comparable with a majority class classifier in the hard case validation. On an external test set we obtained a clearly better performance than a majority class predictor with C5 and SVMs with an RBF kernel. As expected, the performance can be improved by features describing the active site of the kinases by local alignment scores for C5 or position specific features for SVMs. These features are frequently used by C5 and increase the prediction accuracy substantially. Primary chemical features and a diversity in the feature sets had a positive influence on the performance of the classifiers. Note that once a pair of a kinase and an inhibitor is classified as binding, a regression model could be applied subsequently to predict the quantity of the binding affinity.

In summary, the contributions of the paper are as follows: First, we presented a machine learning approach to modeling the binding affinity of inhibitors to kinases. In particular, it is the first time that the complete dataset of Fabian *et al*. [7] with information for all pairs from a set of inhibitors and a set of kinases, is used in predictive modeling (classification). Second, we proposed novel validation schemes for this kind of problem, depending on how much information is available for the inhibitor and for the kinase. Third, our experiments showed that for the decision tree learner C5 alignment-based features are very useful, but that a combination with position-specific features and certain chemical features is necessary for obtaining the best results. For SVMs, the best results are obtained with a quadratic kernel and position specific features. The best predictive accuracy of 86.1% indicates that machine learning methods are able to detect a signal in the data and predict binding affinity to some extent. However, it is clear that there is ample room for improvement for all kinds of methods and that the prediction of kinase-inhibitor binding will remain a relevant research topic for a long time to come.

## Competing interests

The authors declare that they have no competing interests.

## Authors' contributions

FB implemented the methods and conducted the experiments. FB, LR and SK made substantial contributions to the conception, design and coordination of the study. All three analyzed and interpreted the results, were involved in drafting the manuscript, revised it critically for important intellectual content, and gave the final approval of the version to be published.

## Authors' information

FB is a PhD student at the computer science department of Technische Universität München. After receiving his diploma in bioinformatics from the Ludwigs Maximilians Universität München and Technische Universität München, he began to work on predictive toxicology in the scientific staff of Prof. Stefan Kramer (SK) in the Machine Learning and Data Mining in Bioinformatics group at Technische Universität München. His current research interests include predictive toxicology, machine learning, data mining, bioinformatics and cheminformatics.

LR is a Post-doc in the group of SK. He received a diploma and a Ph.D. in biology from Technische Universität München. After a year in a biotech-startup company he came back to Technische Universität München for a postgraduate study in computer science and joined SK's group upon completion. He is interested in data mining and integration of chemical and biological data.

SK is professor of bioinformatics at the computer science department of Technische Universität München. After receiving his doctoral degree from the Vienna University of Technology, he has spent a few years as an assistant professor in the Machine Learning lab of the University of Freiburg. He was the co-organizer of the Predictive Toxicology Challenge 2000-2001, an international competition in toxicity prediction. He has organized several conferences and workshops, edited special issues of journals, given invited talks and tutorials, and serves on the program committees of major data mining and machine learning conferences and on the editorial board of the Machine Learning journal. His current research interests include data mining, machine learning, and applications in chemistry, biology and medicine.

## Supplementary Material

Additional file 1 **In this additional file we show in a table how often an inhibitor binds to a certain group of kinases (group in a phylogenetic meaning)**. It can be clearly seen that nearly all inhibitors bind to several kinase groups. This means that there is generally no kinase group to that an inhibitor binds consistently. Click here for file
