# Supplementary material for: Predicting a small molecule-kinase interaction map: A machine learning approach
Source: J Cheminform. 2011 Jun 27;3:22. doi: 10.1186/1758-2946-3-22 (PMC3151211; doi:10.1186/1758-2946-3-22)
Supplement: Additional file 1 — In this additional file we show in a table how often an inhibitor binds to a certain group of kinases (group in a phylogenetic meaning). It can be clearly seen that nearly all inhibitors bind to several kinase groups. This means that there is generally no kinase group to that an inhibitor binds consistently. [file 1758-2946-3-22-S1.PDF]

Additional file 1 of the paper “Predicting a Small Molecule-Kinase Interaction Map: A Machine Learning Approach”

Fabian Buchwald, Lothar Richter, Stefan Kramer*

Institut für Informatik, Technische Universität, München, Boltzmannstr. 3, 85748 Garching bei München, Germany

Email: {buchwald, richter, kramer}@in.tum.de;

*Corresponding author

Table 1: In this table it is shown how often an inhibitor binds to a certain group of kinases (group in a phylogenetic meaning). From these results it can be clearly seen that nearly all inhibitors bind to several kinase groups. This means that there is generally no kinase group to that an inhibitor binds consistently.

	**AGC**	**CAMK**	**CK1**	**CMGC**	**STE**	**TK**	**TKL**	**OTHER**
**Staurosporine**	5	19	2	11	13	38	2	14
**SB202190**	0	0	1	6	1	3	1	2
**SB203580**	0	0	1	6	2	3	1	2
**VX-745**	0	0	0	2	0	15	0	0
**BIRB-796**	0	1	0	6	3	17	0	1
**SP600125**	3	10	2	8	4	2	0	10
**Gleevec**	0	1	0	5	0	8	0	2
**Iressa**	0	4	1	2	2	6	1	2
**Tarceva**	0	1	0	1	2	7	1	5
**ZD-6474**	0	4	1	0	5	31	1	2
**CI-1033**	0	2	0	2	4	23	1	4
**GW-2016**	0	0	0	0	2	2	0	0
**EKAB-569**	2	7	1	5	8	25	0	8
**Vatalanib**	0	0	0	0	0	5	0	0
**SU11248**	5	17	0	4	7	29	0	12
**MLN-518**	0	0	0	4	2	4	0	0
**LY-333531**	3	8	0	2	6	6	0	7
**BAY-43-9006**	0	2	0	5	3	21	2	2
**Roscovitine**	1	0	3	6	0	0	0	1
**Flavopiridol**	2	8	0	6	0	2	0	4
